# Possibilities for the future of global mental health: a scenario planning approach

**DOI:** 10.1186/s12888-019-2381-3

**Published:** 2019-12-11

**Authors:** Stefan Priebe, Álvaro Arenas Borrero, Victoria Bird, Alma Džubur Kulenoviĉ, Domenico Giacco, Carlos Gómez Restrepo, Fahmy Hanna, Sandrasagary Jayacodi, Seggane Musisi, Craig Morgan, Noeline Nakasujja, Alina Sabitova, Stephen Sandford, Nelson Sewankambo, José Miguel Uribe Restrepo

**Affiliations:** 1 Unit for Social and Community Psychiatry (WHO Collaborating Centre for Mental Health Services Development), Newham Centre for Mental Health, Queen Mary University of London, E13 8SP, London, UK; 2 Department of Social and Preventive Medicine, Pontificia Universidad Javeriana, Bogotá, Colombia; 3 Clinical Center University of Sarajevo, Sarajevo, Bosnia and Herzegovina; 4 East London NHS Foundation Trust, London, UK; 5 WHO Department of Mental Health and Substance Abuse, Geneva, Switzerland; 6 School of Medicine, Makerere University College of Health Sciences, Kampala, Uganda; 7 Institute of Psychiatry, Psychology & Neuroscience, King’s College London, London, UK; 8 Astana Medical University, Astana, Kazakhstan

**Keywords:** Global mental health, Future scenarios, Scenario planning, Global health research

## Abstract

**Background:**

Global mental health is a widely used term describing initiatives in policies, research and practice to improve the mental health of people worldwide. It has been gaining momentum over the last 10 years, reflected in increasing funding opportunities, training programmes, and publications. In light of the rising importance of global mental health and the various uncertainties about its future directions, this paper explores what the future may hold for global mental health in 30 years’ time.

**Method:**

A scenario planning method was used, involving a workshop with experts from four continents and a range of backgrounds, including clinical and academic psychiatry, psychology, art and music therapy, service user advisory role, funder of global health research and post-graduate students.

**Results:**

Six distinct scenarios that describe potential future situations were developed: *universal standards for care; worldwide coordination of research; making use of diversity; focus on social factors; globalised care through technology; mental health as a currency in global politics.*

**Conclusions:**

These scenarios consider different social, economic, scientific and technological drivers and focus on distinct aspects. Some reflect a global application of possible trends in mental health, whilst others apply general global developments to mental health care. They are not fixed forecasts, but instead may help to promote discussion and debate about further developments and decisions.

## Background

Global mental health broadly aims to improve the mental health of people worldwide through various areas of research, study and practice [1]. It is characterised by cross-country interactions in healthcare development and research, usually in the form of collaborations between institutions in high-income countries (HICs) with others in low- and middle-income countries (LMICs).

Over the past decade, there has been an increase in funding opportunities for global health activities, reflected in research initiatives such as the Global Challenges Research Fund (£1.5 billion). This has been accompanied by a growing interest in global mental health issues from a range of stakeholders, notably mental health professionals and researchers, with increasing publications, education and training programmes on this topic. Some have argued that global mental health has emerged as a distinct field within the broader concept of global health, with an emphasis on mental ill health in LMICs [1].

Global mental health tends to encompass a wide range of ideas, approaches and concrete initiatives. Specific activities can investigate mental health indices across countries (e.g. through international partnerships) or examine global influences on mental health (e.g. the impact of climate change). Such activities often consider the economic, social and political contexts in countries, larger regions or worldwide. Given that the context of globally relevant factors will continue to change and that concepts like global mental health are likely to evolve over time, the question arises as to what future there is for global mental health in about 30 years’ time, i.e. the equivalent of one generation from now. The current study is part of funding awarded by the National Institute of Health Research (NIHR) in the United Kingdom (UK) to establish a NIHR Global Health Research Group. One of the aims of the Research Group is to explore and advance understandings of global mental health. The scenario planning study presented here, therefore, aimed to explore potential directions for the future of global mental health to encourage discussion and guide further research.

## Method

Scenario planning is an established method of conceptualising the future through exploring different options for a concept, field or organisation in a specified time horizon [2, 3]. The process creates a number of scenarios that describe hypothetical future situations. The aim is not to produce accurate or most likely forecasts, but instead, embrace uncertainty, challenge current thinking and encourage different ways of conceiving future options. The method has been increasingly used in healthcare contexts [4] and has been applied to other global health challenges, i.e. HIV/AIDS in Africa [5], academic medicine [6], healthcare delivery [7–9] and social perspectives of mental health care [10]. Recently, it was used to speculate on the future of global research [11, 12]. Scenario planning begins by considering current instabilities within a specific context and potentially influential external drivers for change. These instabilities and external drivers for change are then used to construct alternative scenarios, usually with experts in a workshop setting, through a process of discussion and creative thinking.

### Interviews

As a starting point to the current exercise, and for the specific purpose of guiding discussions in the Scenario Planning workshop, we interviewed experts in the field of global mental health to explore potential current instabilities and drivers that could affect the future of global mental health. The findings of the interviews were not intended to reflect representative views but to inform and stimulate the subsequent workshop. We therefore conducted interviews with a range of purposively selected experts, who had either personal experience of practical or research activities in global mental health or an explicit interest in the related concepts. Participants were sampled using the following criteria: working in different world regions and countries including HICs and LMICs, and representing different types of backgrounds and expertise. The experts were identified through existing professional networks, web searches of the literature on global mental health and on-line networks (e.g. Mental Health Innovation Network). Every expert who declined or accepted invitation to be interviewed was asked for further experts who had published peer-reviewed papers related to global mental health or had been involved in global mental health projects. The interviews were conducted either at the Unit for Social and Community Psychiatry, London or via Skype, and followed a semi-structured interview schedule, designed to be open-ended to allow participants to guide the direction of the interview. Topics included current challenges in global mental health, what changes there may be in the world in 30 years, and how this might influence or challenge the direction of global mental health in the future. All interviews were audio-recorded, transcribed, and analysed using thematic content analysis, and key themes were identified.

### Workshop

The key themes from the interviews were presented and informed the scenario planning at a two-day workshop in London. The participants for the workshop were purposively selected to represent different continents, different age groups, a range of backgrounds, and different levels of seniority and expertise. All of them were personally contacted and invited. None of the attendees of the workshop had previously participated in the interviews.

The first day of the workshop was held in an art gallery, the second one in the premises of a research unit. During the whole workshop, free thinking and the shaping of ideas in a group process were encouraged without any requirement for wide consensus on the emerging ideas and scenarios. This was stimulated and supported through arts-based experiential activities. These included: participants visited an exhibition of installations showing clashes of nature with modern civilisation (‘Theatre of the Natural World’) and shared their experiences in pairs; they reflected on their impressions and drafted first ideas in individual drawings; and the themes were then discussed and developed using visual material, including a concept mapping exercise. The preliminary resulting scenarios were then shaped in smaller group work. Each smaller group of participants with an interest in a given scenario developed that specific scenario further. The groups were asked to give the scenario a name and outline a specific example, key actors, drivers and enablers, potential risks and milestones. They were also encouraged to consider possible, preferable and plausible aspects.

The discussion of the emerging scenarios enabled the wider group to specify them and explore alternative scenarios, rather than striving for or assessing consensus.

Following the workshop, the scenarios were further refined, involving repeated discussions in the wider research group of the first author. In these discussions, the core content of the scenarios was not altered anymore, but details were specified focusing on the coherence and explanation of each scenario.

## Results

For the interviews, a total of 108 individuals were contacted, of whom 48 (44%) responded. Eight declined to be interviewed and seven stopped responding, so that 33 experts (31%) were interviewed. Seven experts were interviewed in person, with the other 26 experts the interviews were conducted via Skype. The interviewees included academics (professors, lecturers, research assistants, PhD students), clinicians (psychiatrists, clinical psychologists, nurses) and NGO workers, and were based at LMICs (Brazil, Bulgaria, India, Kenya, Nepal, Nigeria, Pakistan, Palestine, Sri Lanka) and HICs (Canada, France, Ireland, Italy, Portugal, Spain, The Netherlands, United Kingdom, USA).

A wide range of instabilities related to global mental health were put forward. They primarily addressed a disconnect between research and reality, lack of funding and limited research options in LMICs, lack of clarity of what global mental health exactly means and what the related research should achieve, and a dominance of conventional psychiatric concepts and of research led from HICs. Other instabilities included a difficulty to balance social and biological approaches, the lack of coordination of research activities, and a developing ‘research tourism’ serving the interest of researchers in a small number of HICs.

The general drivers for the future of global mental health that were identified in the interviews are summarised in Table 1.

**Table 1:** Drivers for change themes

Drivers for change	
• Advances in technology • Climate change • Rising political instability • Increasing urbanisation • Increasing national economic growth (e.g. middle-income becoming high-income country) • Countries becoming more inward-looking and nationalist • Poorer international relations and reduced international collaboration • Increasing war and conflict • Increasing universality of languages • Increasing migration • Rising role of big data • Widening social and economic inequalities within countries • Other global health challenges becoming more important (e.g. Antimicrobial resistance) • Improved access to education • Increasingly ageing population • Increasingly proactive role of young people in society • Increasing patient and public involvement • Increasing human rights movements • Increasing political prioritisation of mental health issues • General increase in the rapidity of global development and change • Increasing privatisation of health services • Increasing funding for mental health	

The results of the interviews informed the workshop. Twenty participants attended the workshop; attendees were from four continents and consisted of different age groups and included all authors of this paper. Their backgrounds included clinical and academic psychiatry, psychology, art and music therapy, service user advisory role, funder of global health research (the rules of the employing organisation excluded authorship on this paper), and post-graduate students. In the workshop, six different scenarios were developed which were subsequently further refined. The six scenarios are described. The order of the descriptions does not reflect any ranking.

### Universal standards for care

In this scenario, there will be a worldwide consensus about the minimal standards of acceptable professional care for patients with mental disorders. These standards will specify what type of treatment should be provided to all patients in mental health care across the world. The standards will define the regulations and conditions for all built institutions, such as hospitals and sheltered living arrangements, and the precise entitlements of different patient groups to in- and outpatient treatments, social care, and other professional support. This will include standards for involuntary treatment and be driven by a strong human rights movement, partly in response to human rights abuses of patients, particularly those with severe mental illnesses (SMI). The standards will outlaw all forms of mechanical restraint, including chains or shackling of patients, and possibly limit other coercive measures. They will be promoted by a strong patient and carer movement and be agreed on, supported by and disseminated through agencies such as the United Nations, Council of Europe and the World Health Organisation. National and regional mental health legislation will be in line with these standards and guarantee their implementation. A new international organisation or network of organisations will be set up to monitor and report regularly on the implementation of these standards at local levels. The results of the monitoring will be publicly available and the recommendations of the overseeing organisation for local changes will be legally binding. This will lead to a rising proportion of health care funding dedicated to mental health care and facilitate the accessibility of mental health services for all groups in need.

### Worldwide coordination of research

Funding for mental health related research will be coordinated on a global level, potentially through a centralised global research institute. Since most of mental health research will remain publicly funded, this co-ordination will apply to the majority of funds. Supported through better information systems recording all completed and on-going research in the world, the co-ordinating centre will endeavour to avoid duplication of work. There will be no competition between nations as the centre acts in the overall interest of progress in research. There may still be competition between research groups across the world, but the centre will ensure that a limited number of groups work on the same issue at the same time and that very different ideas are pursued with sufficient funding, even if they are not mainstream or fashionable at that time. The main aim of the centre will not be to regulate fairness in the competition between research institutions, but to maximise the impact of research for improving mental health care. This co-ordination will consider or even emphasise specific and unusual local contexts and the situation of people with very rare disorders, but it will do this with a global perspective of priorities and research potentials.

### Making use of diversity

Whilst most current research aims to generate evidence that is generalisable and applies to different populations and places, the emphasis in this scenario will be on understanding and utilising differences to improve practice. Mental health care and research will be focused on embracing diversity rather than striving for similarity and universality, with diversity defined as both the differences between and within countries and regions. It will be accepted that different understandings and treatments of mental distress with equal validity exist across the world, and such differences are the focus of research and inform practice. Anthropologists and qualitative researchers will have a strong role in this. Universal diagnostic tools, such as the DSM and ICD, will be replaced with locally specific instruments and systems, if such normative approaches of instruments are seen as appropriate in the given context at all. International consensus conferences and debates within psychiatry will be replaced by mutually respectful exchanges between those working in this new field. A global repository of research findings, service descriptions and evaluations, and of patient and clinician experiences will be created. All information will be freely accessible to all and further promote mutual learning and understanding of differences. When similarities are observed between certain contexts, this will not be disregarded, but instead offer an opportunity to identify common ground and scope for sharing experiences and learning from one another. Where similar prevalence rates and concepts of a mental health problem are identified, individuals from each context will share effective interventions for this. Overall, this will help to promote multi-directional learning and flexible partnerships across the world to inform the practice of mental health care.

### Focus on social factors

There will be a wide consensus that mental health has strong social determinants. These determinants will become more pronounced and relevant through greater social and economic inequalities in societies across the world. There will be an increased focus on and understanding of such factors, their complex global interconnectedness and their local impact on the mental health of different groups in the population. The production of goods and many services will be easily and rapidly moved to different places, noticeably in socio-economically deprived areas. This will affect all societies around the world, possibly accelerated through developments in information technology. This will quickly and repeatedly change the social conditions in regions across the world and lead to constant migration of large parts of the population. Efforts to improve the mental health of populations will primarily deal with such social factors which can be tackled only on a global level. This will be through poverty alleviation strategies or attempts to reduce rising rates of social isolation in large cities. Generally, there will be a higher prioritisation of mental health issues on a global scale and potentially reduced discrimination against those with mental health problems. This will be because mental disorders are viewed through a wider social lens, rather than regarded as individual and abnormal characteristics.

### Globalised care through technology

Rapid advances in technology will facilitate the delivery of mental health care remotely or virtually through the internet. Assessments and therapies will be delivered from anywhere in the world and, regardless of where people live; all will have access to the same technology-assisted interventions. Some of these interventions will remain steered by professionals and others will be fully delivered through artificial intelligence. In the latter case, mental health support will be available at any time, at any place and potentially at very low costs. The support provided to individuals will remain personalised, but the overall system and type of support will be globally homogenised. One diagnostic tool will likely be used by everyone where algorithms produce a recommended treatment for the individual, potentially self-administered on a smart phone. In response, medication will be delivered remotely through 3D printing and drone delivery. Overall, the majority of mental health care will be truly globalised with the entire global population using the same software programmes to address their mental health problems. There will still be a place for the local provision of mental health care through professionals, e.g. in emergency situations, inpatient care and outreach activities. Yet, this will be substantially diminished and may – over time and at least in part – also be replaced by robots with artificial intelligence.

### Mental health as a currency in global politics

In this scenario, the mental health of populations will become a major currency in the global political discourse and the target for related political actions. Indices of mental health will be regularly measured around the world. The indices will influence or even dominate debates about problems and responses both in general throughout society and specifically among politicians. On a global level, they will be used to assess and compare the success of nations and wider regions. They will have a particular role in the international understanding and dealing with crisis situations. Natural and man-made emergencies will be understood in terms of their impact on the mental health of the affected populations. Different types of difficult contexts such as food and water shortages, armed conflicts with forced displacements, and mass unemployment will all be termed as public mental health crises, and subsequently require actions to improve the mental health of the population. This will apply within nations, but be more important on a global scale, since most major crises will directly or indirectly affect several wider regions and be responded to on a global level. Thus, in sessions of the General Assembly of the United Nations, speakers will argue about suggestions to address mental health crises and to improve the mental health of regions and worldwide. In this scenario, the term mental health problem will have a very wide and relatively consistent global meaning, encompassing most forms of misery, as well as unhappiness and dissatisfaction with life.

The main features of each scenario, consequences for research, health care and governance, the key drivers and potential concerns and implications are summarised in Table 2.

**Table 2:** Summary of scenarios

	Universal standards for care	Worldwide coordination of research	Making use of diversity	Focus on social factors	Globalised care through technology	Mental health as a currency in global politics
Main features	Universal standards for the treatment of patients with mental health disorders	All research activity is coordinated on a global level	Mental health care and research is focused on embracing diversity rather than striving for similarity or universality	Global consensus on social determinants of mental health	Remote and virtual delivery of mental health care	Situations traditionally requiring political action are understood in terms of mental health
Healthcare	Adaptation of local procedures to new set of worldwide standards; Regular audits	Increasingly evidence-based care meeting local needs	Both the organisation and content of care is developed out of or adapted to the local context	Public mental health services providing social interventions; Worldwide community tackling underlying social factors on a global and local level	All treatment can be delivered remotely or virtually, leading to redundancy of services	Increased funding and availability of public mental health services; Clinicians act as consultants for policy-making and government decision-making
Research	Research procedures also adapt to new standards, e.g. informed consent procedures	Centralised management of all research funding and activity; More innovative research; No duplication	Localised research that aims to explore specific and unique aspects rather than striving to produce generalisable evidence; Qualitative methods	Focused on exploring associations between wider social and economic factors and mental health on a global and local scale	Research focuses on developing new technology-assisted diagnostic tools and interventions	Public health and prevention focus
Governance	Global intergovernmental organisations e.g. United Nations	Global governance through centralised research organisation	Local leadership driven by mental health professionals	Greater role of social services and local communities; National and regional governments	Multi-national organisations; Private health firms; Technology companies	National governments and international organisations
Key drivers	Increasing human rights movement; Increasing funding for mental health services; Increasing universal access to information (e.g. through information technology)	More efficient use of funding for mental health research; Increasing research accessibility in LMICs; Advances in technology; Rising role of big data	Increasing national economic growth; Increasing funding for mental health service development	Rapid social and economic change;Increasing urbanisation; Increasing migration; Widening inequalities within countries	Rapid increase in global development; Advances in technology; Stronger role of virtual relationships	Political instability; Climate change; Increasing war and conflict; Increasing prioritisation of mental health issues
Potential concerns and implications	Reliant on one concept of human rights and consensus on standards being accepted universally	Dependent on the views of the central committee members, Potentially strengthening mainstream approach rather than overcoming it; Management of all research worldwide may be difficult to manage in practice	Differences may be so great that there is no scope for learning across contexts (i.e. highly unique healthcare in different settings)	Requires government support and working across different organisations and agencies that may be difficult to bridge in practice; May clash with other political priorities	Potentially less creative, innovative and personalised approaches in mental health care (i.e. replaced by algorithmic software); Potential to drive people further apart through reduced need for face-to-face interactions	Potential neglect of more severe mental disorders; Potential trivialisation of mental health distress

## Discussion

Six scenarios of global mental health in 30 years’ time were developed. They are distinct, but also demonstrate areas of overlap. Some of the scenarios focus more on research than clinical practice, and vice versa, and the likely key actors in each scenario differ. A few of the scenarios, such as *making use of diversity* and *worldwide coordination of research*, illustrate a stronger role of clinicians and researchers, contrasted by their decreasing role in *globalised care through technology*. To different degrees, the scenarios can also be applied to potential futures of the wider concept of global health. On the one hand, *universal standards for care* and *globalised care through technology* could be seen in the context of care delivery for all non-communicable diseases, and *worldwide coordination of research* could be extended to include all global health research. On the other hand, *making use of diversity* may be more specific to mental health, given the various cultural concepts of mental distress and the influence of different social contexts on understanding it [13–15]. Another key difference between the scenarios is the degree of universality. Some scenarios imagine that one universal understanding is accepted worldwide, such as *focus on social factors* and *universal standards for care*. Alternatively, *making use of diversity* and *worldwide coordination of research* can favour more local or culturally-specific approaches. The scenarios differ in the extent to which they may appear appealing or desirable, also demonstrated by the potential concerns in Table 2, yet they all retain an element of plausibility. Moreover, whilst the scenarios were envisaged to develop over the next 30 years, one may argue that some of them such as *universal standards for care* and also *globalised care through technology,* albeit not yet supported through artificial intelligence, have already begun to emerge.

The scenario development method encourages participants to use their imagination and fantasy, and the results inevitably are influenced by personal and situational factors. The method of scenario planning involves a creative process which may not be necessarily replicable in another context, and different scenario planning exercises might come up with different scenarios. However, the six scenarios presented here address various distinct aspects and are likely to cover a range of potential future developments. Future research, could develop these scenarios further, such as assessing whether a wider group of experts share these viewpoints.

What unites the six scenarios is the focus on mental health issues within a global context. One may argue that the scenarios reflect more *universal* than *global* mental health, as the majority of the scenarios move away from a focus on differences between countries and also consider differences within countries. This contextualises the scenarios in a broader, and potentially plausible, world scenario that emerged frequently in the interviews and scenario planning process. That is that economic and social differences between countries disappear, or at least are substantially reduced, whilst they may increase within countries. As such, the distinction between HICs and LMICs may weaken over time. Global mental health must respond to the mental health impact of such changes and not be confined to certain countries or regions. Furthermore, none of the scenarios just extrapolated the current understanding of global mental health, which largely reflects projects funded in HICs focusing on research or practical developments in LMICs. This potentially suggests that the currently widely used term itself may not have a long term future or that different understandings of it will develop.

## Conclusions

The six scenarios presented in this paper are not predictions for the future, but rather speculations. They touch on and may have implications for different fields such as international politics, global ethics, international development, human rights and science more generally. It is likely that none of the scenarios will materialise exactly as envisaged in this study. The future may contain only some aspects of one of the scenarios or combine aspects from different scenarios. However, what all of the scenarios demonstrate is that influencing the future will require debates and decisions that go far beyond the community of experts in mental health, instead wider society needs to be involved – potentially on a global level.

## Data Availability

The datasets used and/or analysed during the current study are available from the corresponding author upon reasonable request.

## Abbreviations

DSM: Diagnostic and statistical manual of mental disorders
HICs: High-income countries
ICD: International classification of diseases
LMICs: Low- and middle- income countries
NIHR: National institute for health research
SMI: Severe mental illness
